# *Bacillus* Strains as Increased Soil Fertility and Biomass Yield Tactics in a Reclaimed Tidal Flat, Saemangeum, Korea

**DOI:** 10.1007/s00284-025-04446-0

**Published:** 2025-08-25

**Authors:** Jihwi Jang

**Affiliations:** 1 Department of Environmental Horticulture, University of Seoul, Seoul, 02504 Korea; 2 Present Address: School of Biological Sciences, University of Canterbury, Christchurch, 8041 New Zealand

## Abstract

The demand for bioenergy resources continues to grow due to increased emerging economic value and emission trading systems. On top of that, energy crop (EC) resources are key to addressing the interconnected global challenges of sustainable development with the adoption of Renewable Portfolio Standard (RPS) and the post-Paris Agreement in Korea. Current research on interactions between EC and plant growth-promoting rhizobacteria (PGPR) emphasizes the energy potential regarding greatly enhanced ecological and economic effects that may increase in the future. However, less attention has been focused on soil fertility management after crop planting in reclaimed tidal land using bio-fertilizers such as rhizobacteria. This review paper documented and evaluated the potential of ECs by discussing the evidence for interactions between PGPR and crops where representative species have grown on Korean reclaimed land during the recent decade. The existing literature revealed several important generalizations that emerge from this synthesis of mechanisms and interaction. First, EC–PGPR interactions affect the increase of biomass yield on reclaimed land without a decrease in values of soil and crop quality. Second, PGPR *Bacillus subtilis* JS increased the T-N (total nitrogen), P (phosphorus), and chlorophyll contents owing to mineral nutrient solubilization and chlorophyll *a/b* binding protein, and *B. licheniformis* MH48 affected the increase of the T-N and P contents in soil because of N_2_ fixation. In this respect, this scoping review highlighted that short-rotation plantation management through PGPR in reclaimed tidal land may be more sustainable than chemical fertilizer treatment in the context of land-use change.

**Supplementary Information:**

The online version contains supplementary material available at 10.1007/s00284-025-04446-0.

## Introduction

### Rationale of the Energy Crops (ECs) Study and the Scope of this Study

Land use and cover change (LUCC) from treeless sites, barren lands, or unused lands such as reclaimed land and landfills to forests, defined as afforestation, has been proposed as one of the most important means to mitigate anthropogenic carbon dioxide emissions [1, 2].

As a means of obtaining energy output, short-rotation coppice (SRC) plantations can offer several advantages.

According to the previous study on the review of SRC management strategy, the application tactics have been more specified [3] (Table 1). Many studies have reported that the optimized SRC methods using poplar trees in various site characteristics range from 1 to 5 years rotation, showing the successful plantation method for the large amount of biomass production over a short period [4], including reclaimed tideland [2, 5]. Indeed, *Populus* has been selected as SRC planning in Korean reclaimed tidal flat (i.e., reclaimed tideland, reclaimed tidal land and reclaimed land) due to the listed reasons. (1) Easily adaptable to a new environment, (2) fast-growing characteristic, (3) it has been used to purify the land with its wide rhizosphere and high transpiration capability from the fertilizer contamination [6], (4) its flourishing rootlet development which facilitates absorbing more moisture and ingredients, and (5) it enhances the quality of soil by providing a better environment for soil microbe [7, 8].

**Table 1:** Management strategies and current applications of poplar from short-rotation plantation [3]

Management strategies	Short-rotation coppice (SRC)	Short-rotation forest (SRF)
Tree density (stand ha^–1^)	≥ 1500	500–1500
Plantation rotation (years)	2–5	8–20
Productivity (Mg^a^ ha^–1^ year^–1^)	Up to 25^b^	6.1–16.3
Rate of harvest (%)	Up to 100	30
Utilization	Heat and power	Pulpwood, saw log, lumber
Price (USD Mg^–1^)	NA^c^	50–120

^a^Dry mass

^b^Leafless total coppice

^c^Means not available for feedstock market price

This study is a scoping review of the literature that addresses (a) the relationship between EC yield and the environmental and site factors of Saemangeum reclaimed land, (b) determinants of biomass yield increment among PGPR and species, and (c) current reclaimed land EC research based on a case study of Saemangeum.

For a better understanding of how main ECs (i.e., poplar, camellia, and kenaf) are cultivated on reclaimed land and forest in South Korea and PGPR (*Bacillus* strains) are used for some species on SRC or plantation management, during the recent decade, we compiled 100 publication that reported data on biomass yield in South Korea and/or utilization bioenergy (wood pellet or biodiesel) and related data regarding the interaction between PGPR and ECs.

### Importance and Growth of Energy Crops (ECs) in Korea

The PGPR inoculation into EC in Saemangeum reclaimed land soils could be a promising option. When the Saemangeum reclaimed land was established, high salinity levels were raised as one of the problems that inhibit plant growth, but it was reported that large areas were detected with low salinity levels and showed silty loam soil properties, which has ability to retain high moisture and commonly used in agricultural activities while showing low soil fertility (i.e., organic matter in Saemangeum ranges from 0.48 to 0.98%, while mean organic matter in agricultural area was 2.3%) over time [4].

As a most recent documentation, Lee et al. [9] reported the Saemangeum soil properties extensively, which includes soil pH and electrical conductivity using the 1:5 weight-to-volume (EC _(1:5)_) in 2015. The soil of Saemangeum reclaimed land has high pH and EC, unlike general field soil, and has low-nutrient retention capacity, making it difficult to grow crops. The total survey area was 242 ha, and the pH and EC _(1:5)_ of 242 points were surveyed. Among the total areas (702 ha), 516 ha (73.5%) were below 0.4 dS/m, 70 ha (10%) were between 0.4 and 1.0 dS/m, and 116 ha (16.5%) were above 1.0 dS/m. The average soil pH in the Saemangeum area was 7.5. Generally, the range of 5.5 to 6.5 is suitable for EC growth, and poplar can grow at around 7.5. However, in areas where the EC _(1:5)_ is above 0.4 dS/m, the root absorption capacity may be impaired. In addition, some areas showed more than 2.0 dS/m, which means a negative impact on poplar growth. Therefore, the most difficult aspects of EC cultivation in Saemangeum reclaimed land are likely to be low soil fertility rather than high salinity levels.

Nevertheless, there are still limited available data on comparison studies of various types of soils (i.e., compared with unfertilized and non-inoculated soil) in EC growth. For these reasons, the management of reclaimed land soil through the comparison study of the effects of PGPR with or without the additional effects of fertilization using promising EC in Korean reclaimed land (i.e., poplar, camellia, and hibiscus) as a verification procedure would be pivotal.

Besides, the overuse of chemical fertilizers can cause unanticipated environmental impacts [10, 11]. Even if nitrogen fertilization can provide sufficient levels of nutrients to increase biomass yield. It might induce adverse impacts such as environmental nitrate (NO_3_^–^) contamination/toxicity or salt accumulation amid NO_3_^–^ leaching from soils [12, 13].

If practitioners and foresters can apply inorganic nutrients which can foster the growth of the rhizosphere system, it will help to increase the production of woody biomass. Among bio-fertilizers, plant growth-promoting rhizobacteria (PGPR) can minimize the salinization of the land by improving the level of pH, nitrogen source from nitric acid (HNO_3_) and ammonia (NH_3_), phosphate (PO_4_^3–^), and the amount of exchangeable positive ions of the land [14]. However, most of the previous studies mainly focused on horticultural and food crops based on horticulture and agronomy, and very little research has been done on the effect of PGPR on woody plants as EC production [2]. Interestingly, when *Bacillus* sp. strain known for PGPR applied by Pidiyar et al. [15] is used on poplar and camellia trees planted in SRC or forestry plantations, it made a prominent result on bioenergy production, increasing biomass yield [2], reducing salinity stress and limited nutrients in the soil of reclaimed land [8, 16].

This review investigated and documented available previous research work (i.e., the existing data and literature) on EC’s growth, biomass yield, and plant biochemical characteristics and defense mechanism on environmental stressors in order to 1) describe the fundamental concept of bioenergy study on reclaimed land, 2) to evaluate the benefits of cultivation characteristics and silvicultural system in reclaimed land, 3) to compile the currently available results—the biomass yield, biochemical, and physiological characteristics of the poplar and camellia seedlings that were treated with *Bacillus* strain, PGPR, and 4) to highlight future research directions that are needed to effectively assess the potential of bioenergy study on reclaimed land.

## Main Woody Energy Crops (ECs) and Their Biomass Production in South Korea: Prerequisite as EC Candidate

### Poplar and Its Biomass Production in South Korea

In South Korea, naturally growing poplars are *Populus davidiana* and *Populus glandulosa* under the *Leuce* class (i.e., white poplars, aspens) and *Populus maximowiczii*, *Populus koreana,* and *Populus simonii* under the *Tacamahaca* class (i.e., balsam poplar). Except for *Populus davidiana*, most of the species are found along the Mt. Taebaeksan range, with very low numbers and dispersion levels [17].

New varieties and mutations were developed successfully through breeding by the introduction of Italian poplar (*Populus nigra* var. Italica) and Canadian poplar (*Populus* × *Canadensis* Moench.) since the establishment of the Korea Forest Genetics Institute in 1956 in Suwon City, Korea, after forest devastation by the Korean War in 1953. Also, *Populus davidiana* Dode × *Populus alba* (*Populus tomentiglandulosa*), *Populus alba* × *Populus glandulosa*, *Populus nigra* × *Populus maximowiczii*, and *Populus koreana* × *Populus nigra* var. Italica were promoted through breeding by hybridization, and aspen was introduced through selection breeding [18]. Later on, the research was continued on a selection of suitable sites [19], which enables us to judge the poplars' natural sustainability without soil analysis and the breeding between non-intersectional cross-incompatibility poplars and pollen compatibility knockout mentor pollen poplars [20].

Research related to forest biomass and bioenergy in young and mature stands is continually in progress. However, the study on the SRCs is still on an unsatisfactory level (not much available data compared with non-inoculated soils) in South Korea. Liesebach et al. [21] reported that they have been examining biomass production using the 5-year and 10-year harvest district aspen SRCs on agricultural land. As a result, they have found that the biomass production from the hybrid aspen was better than that of European and US species, and of 10 years in the harvest district, the biomass production was 100 Mg ha^–1^. Laureysens et al. [22] also reported that they created SRCs on the prior landfill by applying 17 poplar clones and then examined the number of stems to estimate the amount of biomass production. As a result, the average number of stems ranged from 8 to 19 and the average biomass production was 2–11 Mg ha^–1^. Pellis et al. [23] reported that they planted 17 poplar clones with high density and then examined the biomass production and growing characteristics after the first harvest. As a result, the number of stems was 15, the leaf area was 50–60cm^2^, and the biomass production was 3–8 Mg ha^–1^. Labrecque and Teodorescu [24] planted SRCs using 12 kinds of poplar and willow clones along with the unused land and reported that the amount of poplar clone biomass yield was 66.4–72.2 Mg ha^–1^ for four years, where willow clone biomass yield was 62.3–67.5 Mg ha^–1^. A previous report from Kim et al. [4] quantified the biomass production of manure treatment for the year by creating the SRCs with poplars, willow trees, and lilies on abandoned land. The resulting amounts of biomass production for Suwon poplar, willow, and lily were 2.5–3.6 Mg ha^–1^, 2.0–2.6 Mg ha^–1^, and 2.1 Mg ha^–1^, respectively.

Many previous studies mentioned that poplar is suitable for SRC, and they are used for short-rotation harvest in SRC due to their root vigor in barren areas [22, 25] and their fast growth performance [26]. The coppicing method can induce increased forest biomass in intensive silviculture [27].

**Fig. 1:**
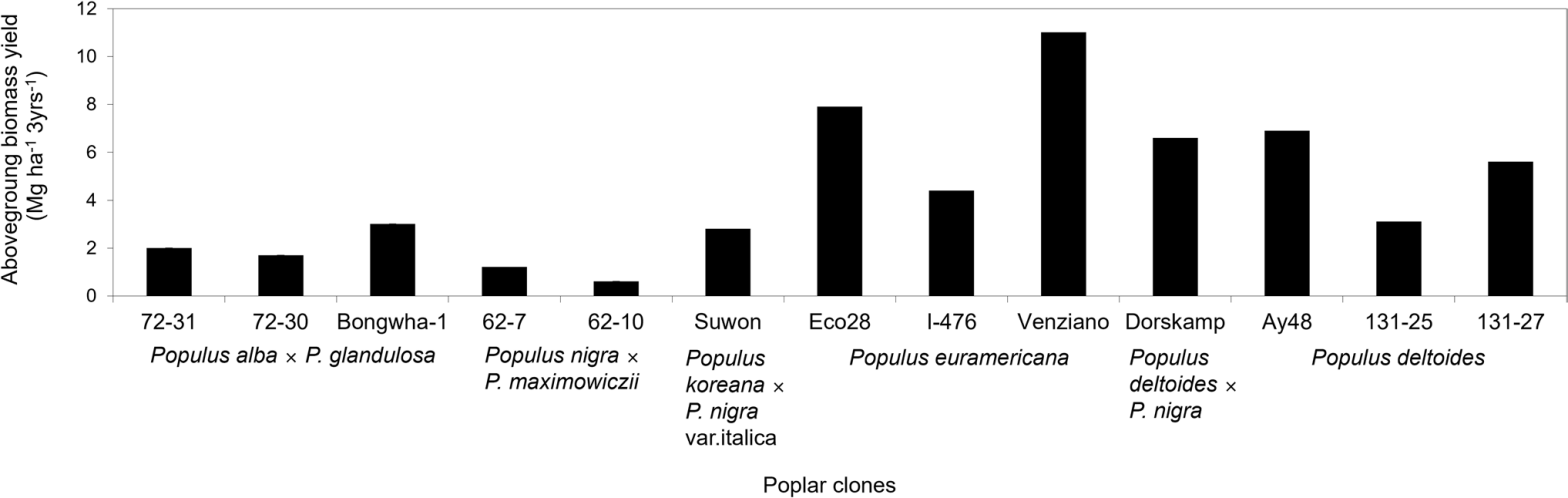
The biomass production after 3-year growing seasons of *Populus* spp. in SRC [30]

EC cultivation and biomass production research have been focused on North American poplar (Canadian poplar and eastern cottonwood), which was introduced to South Korea from the United States are as follows: 1) *Populus* × *Canadensis* Moench. (Eco28, I-476) and 2) *P. deltoides* (Lux). Hybrid poplars developed as new cultivars in South Korea are as follows: 1) Hyun-Sasi (*P. alba* × *P. glandulosa* (Clivus, 72–30, 72–31, Bongwha-1), 2) *P. nigra* × *P. maximowiczii* (62–2), and 3) *P. koreana* × *P. nigra* var. italica (Suwon). In addition, *P. davidiana* was also designated after selective breeding between *P. deltoides* × *P. nigra* (Dorskamp) and *P. deltoides* (Lux) × *P. deltoides* (97–19) [28, 29]. Especially the clones of Canadian poplar (*Populus* × *Canadensis* Moench.) clone Venziano and Eco28 in Fig. 1 showed the best in biomass production [30].

*Populus* spp. has a well-developed rootlet that is suitable for absorbing water and nutrients in the soil. It also provides good conditions for living soil microorganisms and improves soil condition [7], increasing biomass yield in marginal soil such as reclaimed land [2]. Previous studies have reported that biomass yield (20.3%), photo-pigment (25%), and photosynthetic rate (63%) of *Populus* × *Canadensis* Moench were increased by PGPR inoculation and organic fertilizers in SRC in Saemangeum reclaimed land [2, 16].

Song et al. [31] reported that the chemical composition, polysaccharide, and calorimetric values of *Populus* × *Canadensis* Moench., 4 clones (Dorskamp, Eco28, I-476, Venziano) cultivated in SRC on Korean reclaimed land. All poplar clones grown in reclaimed land had higher lignin content (24.4–38.4%) than those grown in normal soil conditions (17.7–23.7%). According to the results of the analysis of lignin, the hot-water soluble extractive contents of poplar bark were higher than those of heartwood and sapwood (i.e., woody core). This is because higher tannin contents and low-molecular-weight materials existed in the bark [31].

Jang et al. [32] reported that the tree height of I-476 and Eco28 clones of poplar was higher than that of other clones among different soil N fertilization treatments and/or PGPR *B. subtilis* inoculation for a few months (i.e., *Bacillus subtilis* JS bacterial culture diluted 1:50 treatment with compost amendment (i.e., fertilizer charcoal) 300 kg ha^−1^). In another biomass and carbon sequestration investigation reported in SRC study in the Saemangeum area, Jang et al. [5] reported the estimated biomass yield of *Populus* × *Canadensis* Moench. In Saemangeum, SRC was 1.85 Mg per ha (originally 103.07 Mg 55.6 ha^–1^ total SRC area), and volumes of CO_2_ absorption were estimated to be 5.93 Mg CO_2_ ha^–1^ (originally 329.72 Mg CO_2_ 55.6 ha^–1^).

In another study, net carbon assimilation and stomatal conductance of *P. deltoides* hybrid clones were lower than Canadian poplar (*Populus* × *Canadensis* Moench.) in peak growing season (i.e., July–August), and that of *Populus* × *Canadensis* Moench. Venziano clone was superior to the other clones in short-rotation coppice (SRC) in Saemangeum area [33].

### Camellia and Its Biomass Production in South Korea

The Camellia genus includes over 260 species, and camellia species have developed over 20,000 ornamental varieties from *Camellia japonica*. From their beginnings as wild plants growing in China, Japan, and South Korea countryside, camellia species have come a long way as breeding technology and cultivation techniques have advanced [34]. By the middle of the nineteenth century, camellia cultivation was widely spread by plant breeders in many nurseries in Western Europe. *C. japonica*, a broad-leaf evergreen woody plant native to Eastern Asia belonging to the camellia family, has the ability to utilize P from relatively unavailable native P sources [35], and is prized for its beautiful flowers and medicinal properties [36]. *C. japonica* is considered one of the most ecologically important flowering trees in southern coastal areas of Korea in terms of its biodiversity [16].

Despite that, few studies have been reported on the species. The camellia is an acidophilic plant due to phenolic-rich root exudates and low pH characteristics and is characterized by specific rhizospheric chemical substances [37]. It has been reported that camellia generally does not produce seeds, and also vegetative propagation by cutting is limited because of the season-dependent rooting ability (i.e., highly affected by winter dormancy of the cuttings in Europe [36]). Unlike the Europe region, camellia is widely distributed naturally in the warm-temperate region in Japan (Honshu, Shikoku, and Kyushu Islands), China (Zhejiang and Shandong provinces), and the southern and western coastal regions of the Korean Peninsula [16], especially in Jeonbuk State (formerly Jeollabukdo-province), Jeollanam-do province [16], and Jeju province that are classified as subtropical forests [38]. The natural population of camellia has been shown to maintain a higher level of within-population genetic variation than other woody species investigated [39]. Camellia is a sub-canopy tree that often occurs and regenerates in gaps among stands [40], a canopy of foliage occurring underneath the uppermost canopy of the forest, typically consisting of large shrubs or small trees. In coastal regions, where environmental stresses are stronger, it has been known to regenerate mainly by sprouting from the base or stem of trees, because few seedlings are observed [41]. It is reported that the estimation of the carbon stored in *C. japonica* is $280.63 Mg CO_2_ ha^–1^ and annual carbon removals are 6.25 Mg CO_2_ ha^–1^ yr^–1^ in Warm Temperate Forest in Korea [28]. However, the growth characteristics (height and diameter) of *C. japonica* stands distributed in South Korea are not clearly documented yet, except for 40 ha with 1,717 tree density (no. of trees ha^–1^) in Jeju province plantation forests [28].

Park et al. [16] reported that *B. licheniformis* MH48 treatment in *C. japonica* seedlings can increase total N and P contents in Saemangeum reclaimed land soils, with higher auxin detection and chlorophyll contents, and it also fostered N and P uptake by the belowground parts of seedlings. However, little has been conducted on growth effects, biomass yield, and their quantification on PGPR and/or *B. licheniformis* MH48 application in reclaimed land yet (i.e., more extensively explored on pathogen effects of *B. licheniformis* MH48 on camellia growth in reclaimed land).

## Main Non-woody Energy Crops (ECs) and Their Biomass Production in South Korea: Prerequisite as EC Candidate

### Kenaf and Its Biomass Production in South Korea

Recently, Kenaf (*Hibiscus cannabinus* L.) has been regarded as an important source of biomass in South Korea due to a shorter life cycle and lower production cost among the herbaceous biomasses [42]. Kenaf plant is a short-day, annual or biennial herbaceous plant (rarely a short-lived perennial), belonging to a member of the Malvaceae family, growing to 1.5–3.5 m tall and is endemic to Africa [43, 44]. It is evident from history that kenaf originated in Sub-Saharan Africa around 4,000 BC in the Sudan region [45]. The kenaf is widely known for its contribution to the global and regional environment because of the significantly high rate of CO_2_ accumulation [46] as well as well grown in the barren area [47] with high tolerance to Hg, Cd, Cu, and Cr contamination of soil without reduction of its biomass yield [48], absorbing soil pollutants such as high N and PO_4_^3–^ in soil [49]. Besides, it is expected to be an alternative raw material to wood fiber in pulp and paper production to avoid the destruction of forests (deforestation) since the 1990s [50].

This kenaf is a promising raw material in the pulp industry because it is capable of high biomass yield under a temperate climate. In addition, most of the parts (i.e., seeds, leaves, barks and stems) can be pulped by conventional wood pulp production processes [51]. Even if kenaf is tropical in origin, its cultivars are well adapted to a wide geographical and climate range [45], with relatively small care as a requirement, hence it is a versatile plant [44, 52]. Early in the 1950s, kenaf species was utilized for producing textile fibers mainly in the Mediterranean region. After the 1980s, kenaf was cultivated mainly for paper pulp purposes [53]. Since 2003, public attention to kenaf species has moved to the bioenergy sector [54]. It was reported that kenaf showed three to five times more biomass yield per unit area than that of many woody plants for pulp production with equivalent quality [55]. For these reasons, as an alternative EC, kenaf can be included in the existing short-rotation coppices (SRC) schemes, which provide the prospects of obtainability of high biomass yield with low management costs [53]. Kenaf is cultivated in many countries (over 20 nations), and main biomass production is from Asian countries (i.e., China, India, and Thailand, approximately 95% all over the world), whereas its production in Africa is very low [44]. In the case of Korea, RDA of Korea established kenaf plantations for sustainable biomass energy production in 2011. To obtain high biomass yield in plantations, reducing the production cost in large cultivation areas, one of the most relevant areas for intensive cultivation to maximize kenaf biomass yield was Saemangeum reclaimed land in South Korea [52]. Generally, kenaf cultivars are classified roughly into three types as early maturing cultivars, mid-season cultivars, and late varieties in the world [54]. Whereas the early maturing cultivar grown in South Korea has been known to have low biomass productivity, even some cultivars rarely produce their seed yield [52]. Recently, new kenaf cultivars have been developed by mutation breeding using exposed gamma rays, especially in South Korea [56]. Previous studies reported the biomass yield of the kenaf cultivar was 20–27 Mg ha^–1^ in South Korea [52, 56] and net photosynthetic rate of them ranged from 22.38 to 25.46 μmol m^–2^ s^–1^ [57]. The height of kenaf grown in a plantation in South Korea has been reported as 3.84 m (for 200 days) on average, but the growth (height and diameter) of them grown in Saemangeum reclaimed land ranged from 2.48 to 3.68 m and 2.0 to 2.9 cm, respectively [52]. Kenaf is a spotlighted energy feedstock for biofuels production owing to its high cellulose contents for producing bioenergy [44]. However, the adoption and cultivation of kenaf in Korea have been conducted recently, and its feedstock has been used for livestock feed and fiber or textile mainly [52, 57]. While, after the 2010s, kenaf was re-evaluated as a feedstock producing bioenergy such as biodiesel and combustion for energy production and heating, cultivating large plantations such as the Saemangeum area [52]. Saba et al. [44] reported that kenaf has spread well with environmental adaptation, and the average biomass yield in the normal plantation is around 12.35 to 18.53 Mg ha^–1^. For these reasons, it is being considered to manage kenaf on Saemangeum plantation even if it grows in reclaimed land [52]. However, there are no available data and references on the effects of microbial and/or PGPR on kenaf growth in reclaimed tidal land all over the world so far. Therefore, it is required to update the research that can fill out the research question regarding the effect of PGPR application in *Hibiscus* species.

## General Mechanism of Plant Growth Promoting Rhizobacteria (PGPR)

**Fig. 2:**
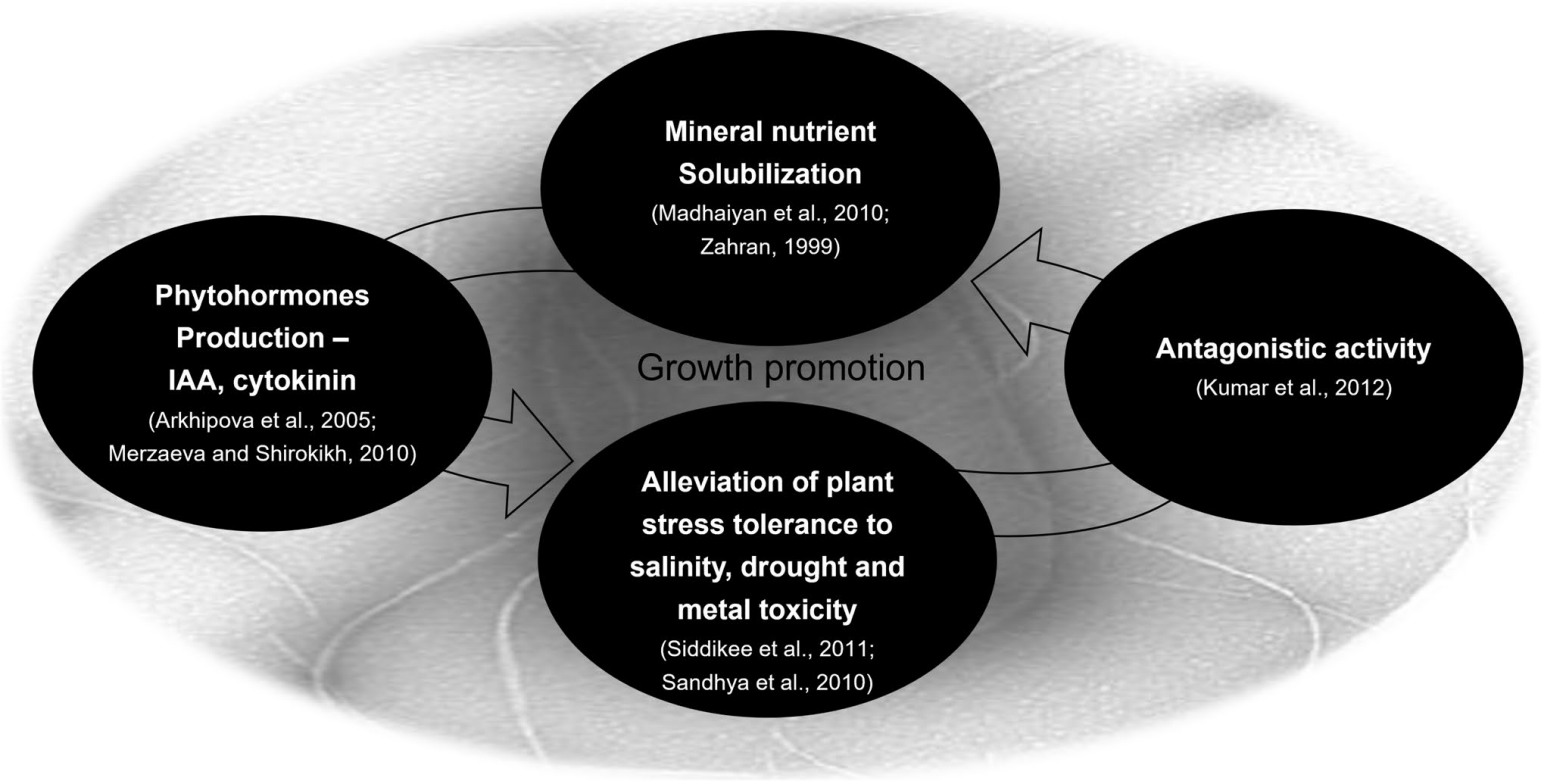
Plant-growth-promoting mechanism of PGPR [59, 61–63]

Plant growth-promoting rhizobacteria (PGPR) are assessed as advantageous microbes to plants for growth promotion. Various bacteria such as *Bacillus* spp. have been reported to foster plant growth through direct and/or indirect mechanisms [58]. The reported conceptual mechanisms of plant growth promotion by PGPR are as follows (Fig. 2): (1) phytohormones production (i.e., auxin, cytokinin, ethylene, and gibberellins) [59]; (2) N solubilization and N_2_ fixation [60]; (3) antagonistic effects against pathogen via chemical substance production such as siderophores, β-(1,3)-glucanase, chitinase, antibiotics, and cyanide [61], (4) P solubilization through rhizosphere [62], and (5) abiotic stresses alleviation such as salinity, heavy metal, and drought stress [63].

## Use of Plant Growth Promoting Rhizobacteria (PGPR) for the Improvement of Energy Crop (EC)

*Pseudomonas* genus, *Bacillus* genus, etc. help to prevent pests and promote plant growth, especially *Bacillus* genus is known as a strong rhizosphere fixative. [64]. Depending on the acreage of the crop grown, there is also the case that the salinity level of agricultural land increases. Therefore, microbial development research is being performed to lower the salt concentration [65]. When *Pseudomonas putida* was processed under salt stress conditions of cucumbers, compared to the control groups, it showed growth in crop yields and an increase in leaf area [66]. In addition, increases in chlorophyll and stomatal conductance are reported after treatment of the *Bacillus* genus, Glomus in lettuce under soil salinity [67].

**Fig. 3:**
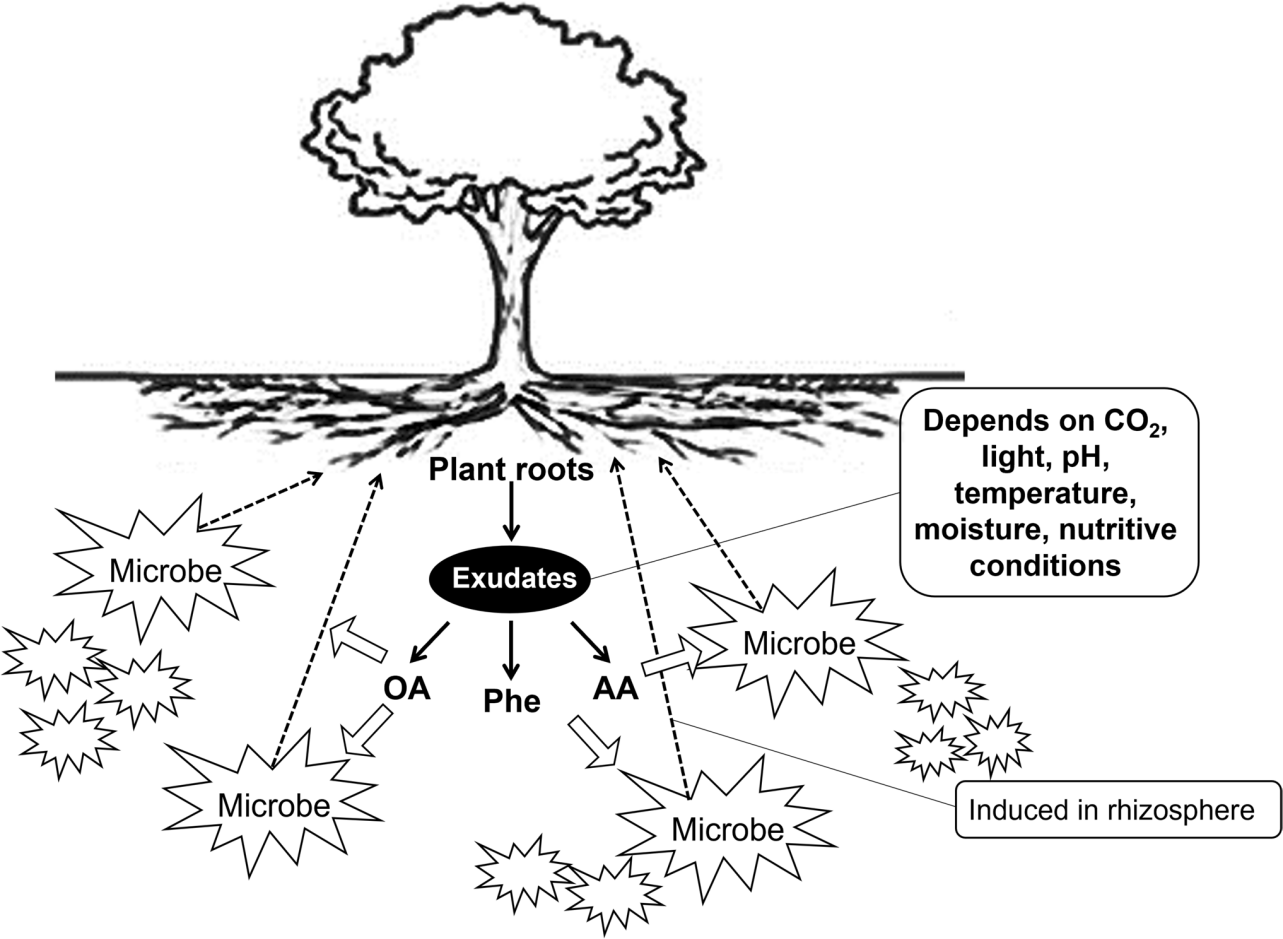
Effect of root exudate on nutrient availability and uptake by rhizosphere microbes. (OA, organic acids; AA, amino acids; Phe, phenolic compounds. Arrow; black, exudation; white, uptake/stimulation; dotted, chemotaxis) [99, 100]

There is a report that rhizosphere physicochemical and biological properties formed by the root exudates of the plants influence the activity of rhizobacteria [68]. For example, if there is insufficient P in the plant, it discharges the exudate, such as carboxylic acid (citric acid, malic acid), to change the pH of the soil, amino acid emissions from the plant roots to stimulate the chemotaxis of soil microbes, and induce into the rhizosphere (Fig. 3).

**Fig. 4:**
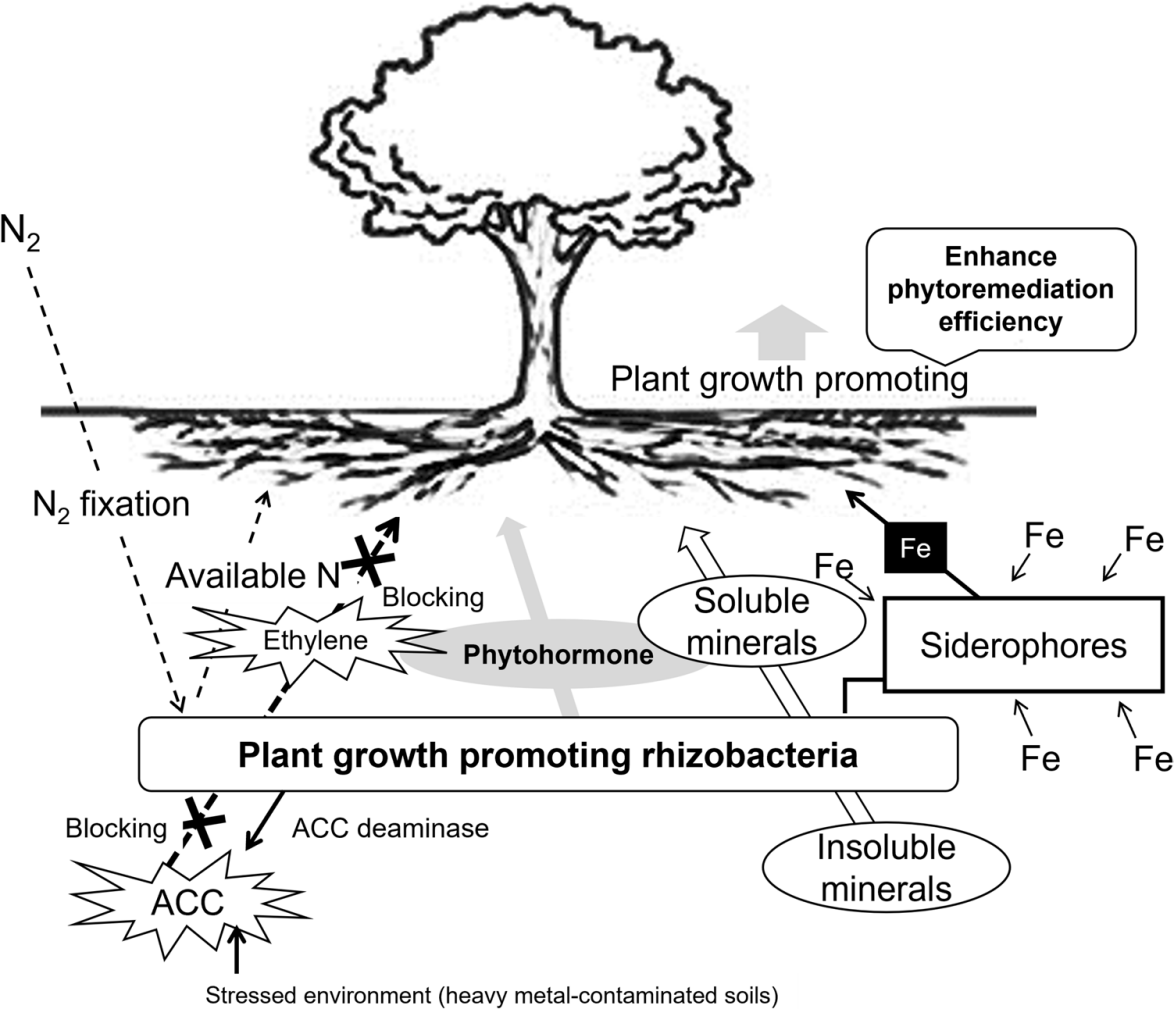
Functions of plant growth-promoting rhizobacteria [100]

Figure 4 shows that the functions of PGPR are well documented, which may serve as a conceptual model to address the effect of plant growth promotion and several uptake kinetics in the root system. Rhizosphere microorganisms live in the roots, and the older roots are denser than the radicles. This is thought to be the result of the root’s large amounts of secreted substances [69]. When *B. megaterium* and *P. fluorescens* are inoculated under in vitro or in vivo conditions, it has been reported that various plant growth-promoting effects under the terms of the cultivated environment [70].

When this type of useful microorganism in the plant is processed, microorganisms 1) dissolve inorganic PO_4_^3–^ using phosphatase, and/or 2) dissolve unavailable P in an organic acid to improve the availability of P in the plant. There is a report that an increase in plants’ nutrient uptake ability is due to the special promoting action on the uptake during the ion absorption process and growth of the root surface area [71]. When seedlings of tomato strains were inoculated with some *Azospirillum*, the occurrence of root hairs significantly increased in the region near 1 cm from the root apex. Then, the root surface area increased. This indicates that it promotes the absorption of plant nutrients [72].

Sarig et al. [73] reported that the water accessibility of the plant improves by treating the plant’s growth-promoting rhizosphere microorganisms during the field experiment. *A. brasilense* inoculation enhances the water availability of sugarcane and increases LWP (ψ_w_). The temperature of the canopy was lowered, and stomata conductivity and transpiration rates increased. In this case, the water content in the soil that was inoculated with the microorganism was about 5% higher than the water content of the soil without inoculation.

According to Kloepper et al. [74], plant growth-promoting activity was highly achieved by limiting the iron absorption and the use of indigenous microorganisms in the rhizosphere. PGPR secretes siderophores (microbial iron transport agents) out of the cell, and this makes it impossible to use the iron of indigenous rhizosphere microorganisms. According to Kim [75], *B. subtilis* JS is a microorganism strain isolated from the rhizosphere soil thatch. Kim [75] has also reported that *Bacillus subtilis* JS markedly increased tobacco seedling growth by emission of volatiles under in vitro conditions. Seedling’s shoot fresh weight, length of primary roots, and the number of roots increased compared with control. Hwang [76] reported that among the microbial agents, especially rhizobacteria promote the growth of plants and pest control of the crops with rapid decomposition, which is faster than chemical pesticides. As a result of *B. subtilis* JS in vitro experiment with volatiles, plant pathogens such as *Fusarium oxysporum*, *Sclerotinia sclerotiorum*, and *Phytophthora capsici* increased the disease resistance of the plant. Secondly, *B. subtilis* JS’s secretion of volatile substances participates in the growth of the sweet potato. The weight of the aboveground part increased, and the growth of the root was facilitated. Park [77] used *Bacillus amyloliquefaciens* KB-MJK 601 strains to determine the antimicrobial activity and plant growth-promoting effect. He diluted the strain to a total of four levels 1 × 10^10^ CFU ml^–1^ 1:50, 1:100, 1:500, and 1:1000. The port is 15 × 18 cm and 500 ml per port. A drench and foliar fertilization of tomatoes had been formally processed every week. One day later, the growth-promoting effect was observed.

PGPR are known to confer resistance to biological stress [78]. Volatile materials such as 2R,3R-butanediol from *Pseudomonas chlororaphis* 06 use stoma closure of the plant through the hormonal changes and reduce the water loss of the plant to give a dry resistance to *Arabidopsis* [79]. Mayak et al. [80] reported the effects of *Achromobacter piechaudii* ARV8 inoculation on tomatoes and capsicum seedlings, which increased seedlings’ fresh weight and dry weight under the short-term water stress state. By reducing the ethylene of plants that receive salt stress, volatile materials can induce resistance to salinity stress and promote plant growth.

Therefore, the volatile materials and exudates that can be caused by rhizobacteria provide promoters for inorganic nutrients such as P, N absorption, and other plant hormones such as IAA and cytokinin, which can be utilized by plant growth.

According to a notable study conducted in the SRF of Korean reclaimed tidal land, specifically in the Saemangeum area, Park et al. [16] reported that the *B. licheniformis* strain MH48 significantly increased the aboveground and belowground biomass of *Populus* up to 15.67 g seedling^–1^ and 8.00 g seedling^–1^, respectively. Jang et al. [2] also reported *B. subtilis* strain JS induced poplar seedling growth and its biomass yield in the Saemangeum reclaimed tidal land, which compares *B. subtilis* inoculated and non-inoculated soil and also combined treatments of *B. subtilis* and compost amendment (charcoal fertilizer) induced 303.33% biomass yield increase with higher growth rate and physiological vigor.

A previous study also reported that in the EC-planted area in the Saemangeum reclaimed tidal land, EC _(1:5)_ was lower than that of the unplanted area due to the unplanted areas' lower water potential, vulnerability to a decrease in precipitation, and an increase in evaporation [9]. In addition, *B. subtilis* affected the change of soil EC levels. After combined treatments of *B. subtilis* and compost amendment in EC-planted areas, EC _(1:5)_ was significantly decreased compared with control (i.e., > 0.4 dS/m) due to the facilitation of water and nutrient uptake [2, 16]. Therefore, the effects on plant growth and development using a particular *Bacillus* strain might be significant for soil condition improvement in this area.

To sum up, putative mechanisms of PGPR to foster biomass and bioenergy production via EC were based on 1) 1-Aminocyclopropane-1-carboxylic acid (ACC) deaminase production by *Pseudomonas putida*, *Enterobacter cloacae*, *Kluyvera ascorbata* SUD 165, 2) IAA production by *Bacillus* spp.*,* 3) Solubilization of phosphorus induction by *Bacillus* spp.*,* 4) Cytokinin production by *Bacillus* spp.*,* 5) 2R,3R-butanediol volatile material production by *B. subtilis* GB03, 6) 2-Pentylfuran production by *Bacillus megaterium* XTBG34, 7) Chlorophyll a/b binding protein, Methionine-R-sulfoxide- reductase B4 protein, and Glutathione S-transferase-parA induction by *Bacillus subtilis* JS and 8) Siderophores and indole-3-acetic acid production by *Kluyvera ascorbate* and SUD 165.

The following Table 2 is the list of the PGPR’s putative mechanisms and functions regarding biomass yield and environmental acclimation so far.

**Table 2:** Influence of plant growth-promoting rhizobacteria on biomass yield

Potential	PGPR	References
ACC deaminase	*Pseudomonas putida*	[81]
	*Enterobacter cloacae*	
	*Kluyvera ascorbata* SUD165	
IAA	*Bacillus pumilus* *Bacillus licheniformis*	[82]
	*Bacillus* OSU-142 *Bacillus* M-3 *Bacillus licheniformis* RC08 *Bacillus megaterium* RC07 *Bacillus subtilis* RC11 *Bacillus cereus* RC 18	[83] [84]
	*Bacillus licheniformis* MH48	[16]
Solubilization of phosphorus	*Bacillus* M-3, *B. brevis,* *B. Cereus, B. Circulans,* *B. firmus, B. licheniformis,* *B. megaterium, B. mesentericus,* *B. mycoides, B. polymyxa,* *B. pumilus, B. pulvifaciens,* *B. subtilis*	[83, 85]
Cytokinin	*Bacillus* OSU-142 *Bacillus* M-3	[83]
2R,3R-butanediol	*B. subtilis* GB03	[86] [87]
2-Pentylfuran	*Bacillus megaterium* XTBG34	[88]
Chlorophyll *a*/*b* binding protein, Methionine-R-sulfoxide- reductase B4 protein, and Glutathione S-transferase-parA	*Bacillus subtilis* JS	[75] [2]
Siderophores, IAA	*Kluyvera ascorbate* SUD 165	[89]

*ACC* 1-Aminocyclopropane-1-carboxylic acid, *IAA* indole-3-acetic acid

## Effect of *Bacillus subtilis* JS and *B. licheniformis* MH48 on Energy Crops (ECs) in Korea

### *Bacillus subtilis* JS

*Bacillus subtilis* is a well-studied aerobacter and also belongs to a member of Gram-positive bacteria [90]. It is defined as the subspecies of *Bacillus* with *B. cereus* according to their capacity to suppress plant pathogens [91]. *B. subtilis* is naturally present in most places, such as soil, water, air, and soil organic matters (i.e., decomposing plant material), while it is not biologically active and presents in the spore form under most conditions. There are two ways of applying *B. subtilis* strains to a plant: 1) foliar application, and 2) soil drench for root application [92].

*B. subtilis* can readily form a circular or oval-shaped spore to endure and survive under unfavorable and stressed conditions. The most known function of *B. subtilis* is plant growth promotion by biochemical mechanisms and its volatile material [75]. Zou et al. [88] reported that *Bacillus megaterium* XTBG34’s volatile materials (2-Pentylfuran) fostered the growth of *Arabidopsis thaliana*. Likewise, Ryu et al. [86] and Zhang et al. [87] revealed that *B. subtilis* GB03’s volatile materials (2R, 3R-butanediol) increased the fresh weight of *A. thaliana* and induced auxin to increase. Also, the antibiotic production of *B. subtilis* triggers plant disease inhibition [93]. Thus, the substance produced by antibiotic production or many secreted hormones by *B. subtilis* is able to facilitate the plant's growth directly and/or indirectly [94].

In Saemangeum area, the study on the interaction between *B. subtilis* inoculation and biomass yield for wood pellet production was first reported by Jang et al. [2], using strain *B. subtilis* JS. Kim [75] reported that rhizobacteria, strain JS (National Patent Classification Code: KR1020140028777), markedly increased tobacco seedling growth (i.e., shoot fresh weight, the number of lateral roots and root hairs, and the primary root length) by emission of volatiles in vitro. *B. subtilis* JS was obtained by reproducing fungi isolated from the rhizosphere of flame grass (*Miscanthus sinensis* var*. purpurascens*) grown in Korea. This strain was chosen because it helped lettuce and tobacco plants survive in harsh conditions of abiotic stress. The isolates and inoculation used in this study followed previously published procedures. The *B. subtilis* JS gram-positive bacterial strain identified by Song et al. [95] was used during the experiment.

Volatiles of *B. subtilis* JS play a role as elicitors. These stimulate the expression of hormone-related genes and chlorophyll a/b binding protein gene, resulting in plant growth promotion. They also affected the increase in the expression of a series of PR genes, conferring disease resistance on plants. Volatiles of *B. subtilis* JS induce a reduction of expression of ROS scavenging genes, indicating ROS stress may result in damage to cells [75].

In the field trial, to investigate whether the effects of PGPR with/without compost amendment (i.e., charcoal fertilizer) in EC (i.e., poplar) are synergistic or not, Jang et al. [2] highlighted that the combined effect of *B. subtilis* JS inoculation and charcoal fertilizer treatment was efficient in increasing biomass yield in reclaimed land (i.e., tillage and fertilization treatment of mixed treatment of charcoal 300 kg ha^−1^ and soil drench *B. subtilis* JS bacterial culture diluted 1:50) was significantly higher than *B. subtilis* JS singly. However, there are no recent updated data available regarding the effects of PGPR inoculation in poplar species in reclaimed land since 2017.

### *Bacillus licheniformis* MH48

*Bacillus licheniformis* strain MH48 (GenBank accession No. KP099612) has been more actively utilized for bioenergy crop pathology study rather than biomass yield and plant physiology in reclaimed land and plantation in Korea [16]. Jeong et al. [96] reported that rhizobacteria *B. licheniformis* strain MH48 demonstrated significant antifungal activity against plant pathogens such as *Rhizoctonia solani*, *Colletotrichum gloeosporioides*, and *Phytophthora capsici*. In addition, it is reported that *B. licheniformis strain* contributes jujube (*Ziziphus jujuba* Miller var. *inermis* Rehder) fruit yield increase by mitigating fungal pathogens and rotting damage caused by it [97]. Won et al. [34] also reported that *B. licheniformis* MH48 inoculation to Coastal Pine (*Pinus thunbergii* Parl.) significantly decreased Fusarium Root Rot (*F. oxysporum*) growth and showed higher seedling growth and biomass yield in inoculation and chemical fertilizer treatment simultaneously. *B. licheniformis* also contributed to an increase in root hair growth and lateral root development for better nutrient uptake through a contribution of IAA production in poplar seedlings (*Populus* × *canadensis* Moench) [98]. Consequently, inoculation of *B. licheniformis* is likely to affect higher bioenergy production by improving the foliar chlorophyll content and carbon assimilation under the antagonistic effect of plant pathogens.

Jeong et al. [96] and Park et al. [16] reported that strain MH48 showed a rapid growth rate from 1 to 2 days after inoculation. After that, it gradually decreased to 7 days. The highest cell growth was found to be 29.7 × 107 CFU mL^─1^ after 2 days of incubation.

In the context of the reclaimed land study, it has been reported that *B. licheniformis* MH48 has a positive effect through controlling foliar fungal diseases, especially antifungal activity against *R. solani* and *C. gloeosporioides* [96] and fostering growth promotion of *Camellia oleifera* [16] and *Pinus thunbergii* seedlings in coastal reclaimed land through fostered nutrient uptake [34]. Won et al. [34] also reported that *B. licheniformis* MH48 can increase T-N (total nitrogen) and P (phosphorus) contents in the soils through N-fixing and P solubilization.

Park et al. [16] reported *B. licheniformis* MH48 inoculation increased the T-N and P content in the reclaimed land soil owing to N_2_ fixation and the P solubilization through *B. licheniformis* MH48’s root exudate (i.e., organic acid). In contrast, chemical fertilization showed limited nutrient uptake and solubilization in camellia seedlings under saline soil of reclaimed land. In addition, limited nutrient solubilization and leaching resulted in low-nutrient contents in the soil and showed limited seedling growth. Besides, *B. licheniformis* MH48 induced the production of auxin, stimulating root development and nutrient uptake. It is also reported that rhizobacteria inoculation reduces the ethylene levels in plants by containing ACC deaminase [81], alleviating salt stress [16].

Also, Park et al. [8] and Won et al. [34] documented that a single treatment of chemical fertilizer was not effective on camellia growth, while combined treatment *B. licheniformis* strain MH48 and chemical fertilizer showed better plant growth with antagonistic effects under pathogen treatment. Nevertheless, there are no recent updated data available regarding the effects of PGPR inoculation in camellia species in reclaimed land since 2019.

**Fig. 5:**
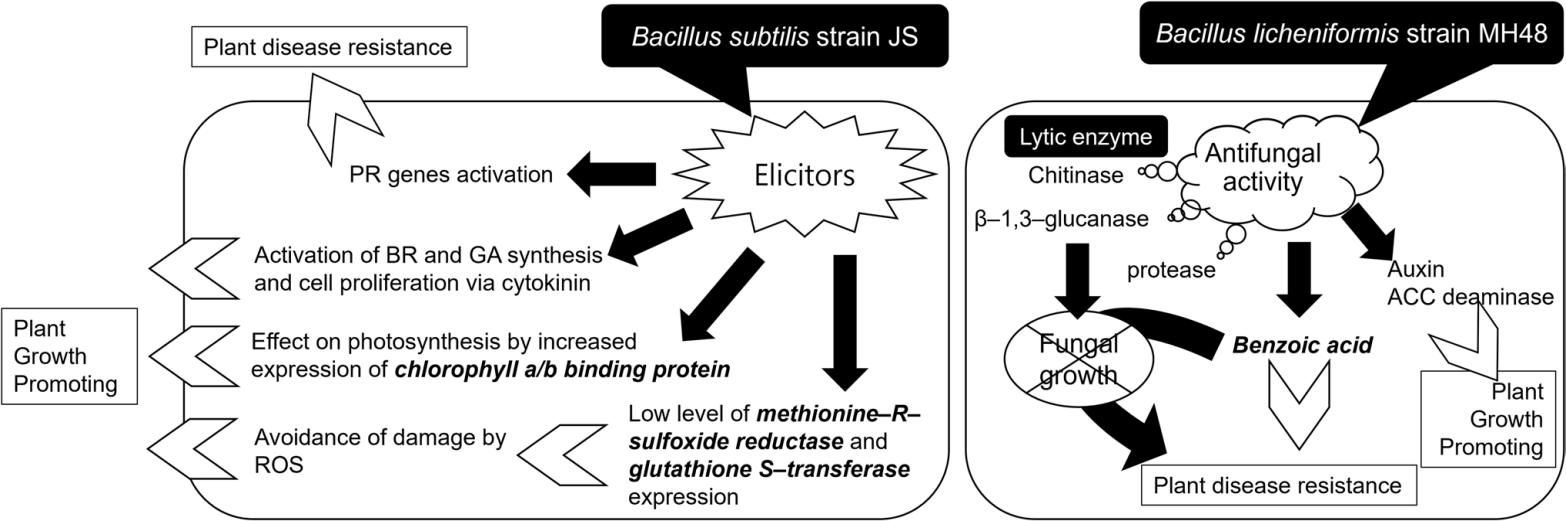
Putative mechanism of growth promotion and enhanced disease resistance on the plant by volatiles of *Bacillus subtilis* JS and *Bacillus licheniformis* MH48 [16]

In summary, Fig. 5 shows the schematic of the putative mechanism of growth promotion on the plant by volatiles of *B. subtilis* JS and B. *licheniformis* MH48.

In Saemangeum area, the studies on the interaction between *B. licheniformis* inoculation and seed yield for biodiesel potential have been conducted on small-scale forest areas rather than afforestation areas. Therefore, it is likely to require long-term monitoring with larger-scale research for future studies.

## Conclusions, Limitation, and Future Research Direction

Interaction mechanisms are classified according to how they influence sustainable biomass yield and soil fertility, and whether the interactions have synergetic effects for applicability. Several important generalizations emerge from this synthesis of mechanism and interaction. First, EC–PGPR interactions affect the increase of biomass yield on reclaimed land without a decrease in values on property and utilization. Second, PGPR *Bacillus subtilis* JS increased the T–N and P, and chlorophyll content of leaves owing to mineral nutrient solubilization and chlorophyll a/b binding protein, and *Bacillus licheniformis* MH48 amplified the T-N and P content of soil owing to N_2_ fixation. Lastly, the inoculation of PGPR with fertilization procedures (i.e., *Bacillus subtilis* JS soil drench treatment (diluted 1 × 10^10^ CFU ml^–1^ 1:50) at the site where compost amendment was treated (i.e., tillage and fertilization treatment of charcoal 300 kg ha^−1^)) showed the optimal biomass yield results and soil EC _(1:5)_ level stabilization compared with PGPR with non-fertilization procedure in Saemangeum reclaimed land according to the case study. In this respect, the concept of short-rotation plantation management through PGPR may be more sustainable than the non-use of PGPR or chemical fertilizer treatment.

The use of marginal lands, such as reclaimed tideland, for SRCs might contribute to a sustainable energy supply in the future, especially in countries that have limited land space due to 62.6% of the mountain forest areas. The adaptability, growth pattern, and management of poplar and kenaf have an essential impact on yield and quality parameters in reclaimed land, especially in Korea. PGPR are crucial microbials that enhance plant growth and biomass production of woody crops in low-nutrient reclaimed land, especially in the Saemangeum area of Korea. Based on these studies, it is expected that coastal short-rotation forest management in Korea will be developed using soil fertility management. Moreover, this knowledge of forest management strategies can be applied to quality and yield management. However, according to reviews of previous studies on biomass yield in reclaimed land, there is little known beneficial effect, and even the study related to this topic (i.e., the interaction between PGPR inoculation and growth on coastal forest and/or reclaimed land forestry). The increased soil fertility using *Bacillus* strain is important to research that results in terms of coastal forest management, improving seedling’s biomass yield and nutrient solubilization in reclaimed land, owing to enhanced soil nutrients and rootlet systems. In this review, it has been confirmed that poplar seedlings planted in the Saemangeum SRC seem to be well adapted to the harsh edaphological conditions of this region under increased soil enrichment of reclaimed land soil. This review also showed that the effects of rhizobacteria *Bacillus* strain JS and MH48 on biomass yield, soil chemical properties, and physiological characteristics were efficient for growing poplar trees in marginal soil, such as reclaimed land, and contribute to wood pellet and biofuel utilization by presenting a method for soil enrichment. However, to update data, regarding no available data on the effects of PGPR in camellia and kenaf in Saemangeum reclaimed land, it would be required to confirm how *Bacillus* strain JS and MH48 affect biomass and bioenergy from camellia and kenaf in Saemangeum reclaimed land in the near future.

In conclusion, here, gathering an unprecedented dataset, the author suggests that the current interpretation of the PGPR needs to be updated to account for the potential roles of PGPR with/without compost amendments in confirming the fast-growing and biomass production in root strategies of EC in reclaimed tidal land.

## Supplementary Information

Below is the link to the electronic supplementary material.

Supplementary file1 (PDF 554 kb)

## Data Availability

Additional data are available following any request.

## Materials Availability

All the datasets are included in the manuscript.
