# Supplementary material for: Bacillus Strains as Increased Soil Fertility and Biomass Yield Tactics in a Reclaimed Tidal Flat, Saemangeum, Korea
Source: Curr Microbiol. 2025 Aug 25;82(10):474. doi: 10.1007/s00284-025-04446-0 (PMC12378901; doi:10.1007/s00284-025-04446-0)
Supplement: Supplementary file 1 — Supplementary file1 (PDF 554 kb) [file 284_2025_4446_MOESM1_ESM.pdf]

Bacillus strains as increased soil fertility and biomass yield tactics in a reclaimed tidal flat, Saemangeum, Korea; Current Microbiology; Jihwi Jang; University of Canterbury; Jihwi.jang@pg.canterbury.ac.nz

Figures in online appendices (Electronic Supplementary Material)

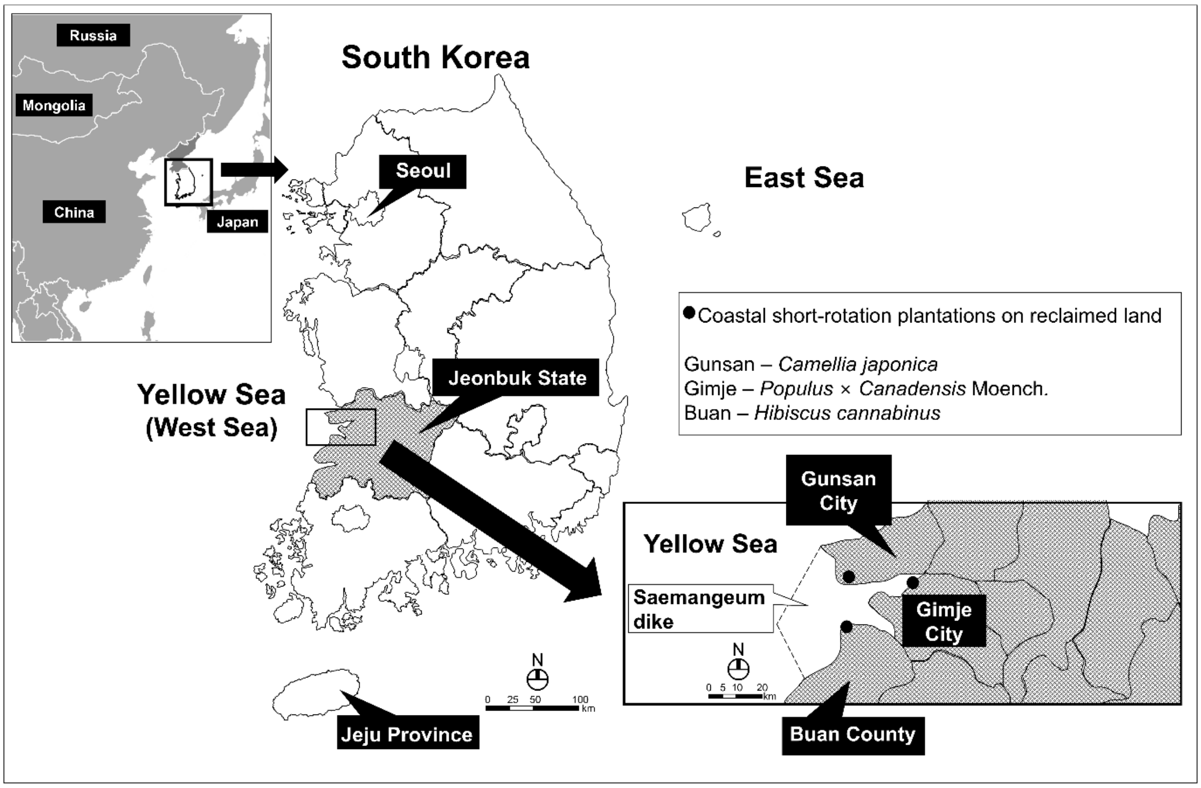

Fig. A1 Geographical information and main energy crops in the Saemangeum area

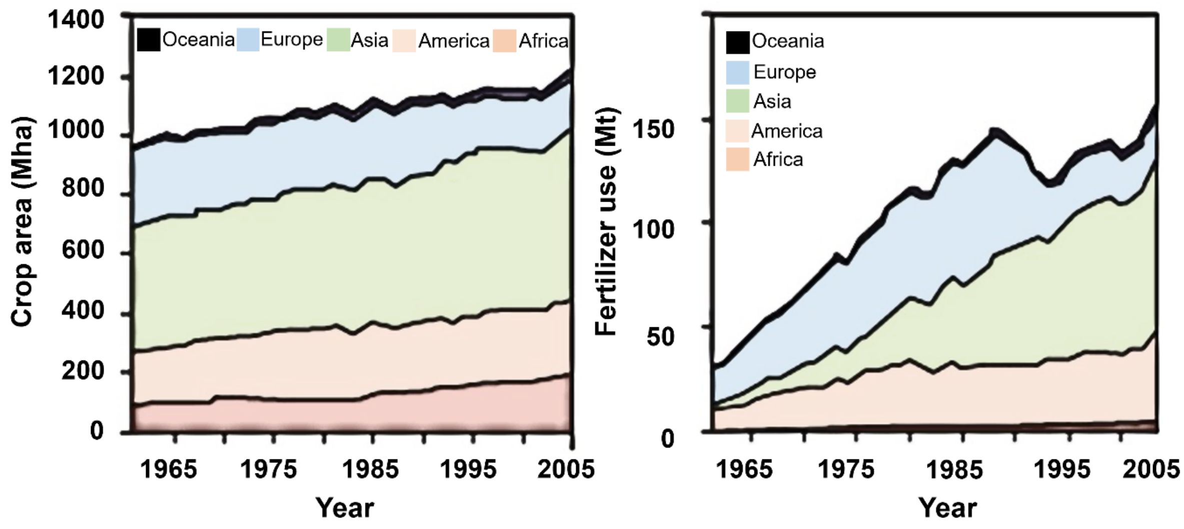

Fig. A2 Farmland area in the world (left) and changes in fertilizer application rate (right) [18, 19]
